# Bio-Inspired Polymer Membrane Surface Cleaning

**DOI:** 10.3390/polym9030097

**Published:** 2017-03-09

**Authors:** Agnes Schulze, Daniel Breite, Yongkyum Kim, Martin Schmidt, Isabell Thomas, Marco Went, Kristina Fischer, Andrea Prager

**Affiliations:** Leibniz Institute of Surface Modification, Permoserstr 15, D-04318 Leipzig, Germany; daniel.breite@iom-leipzig.de (D.B.); gonyyong@live.unc.edu (Y.K.); martin@schmidtf.de (M.S.); isabell.thomas@iom-leipzig.de (I.T.); Marco.Went@iom-leipzig.de (M.W.); Kristina.Fischer@iom-leipzig.de (K.F.); andrea.prager@iom-leipzig.de (A.P.)

**Keywords:** polymer membrane, surface modification, enzyme immobilization, catalytic properties, self-cleaning

## Abstract

To generate polyethersulfone membranes with a biocatalytically active surface, pancreatin was covalently immobilized. Pancreatin is a mixture of digestive enzymes such as protease, lipase, and amylase. The resulting membranes exhibit self-cleaning properties after “switching on” the respective enzyme by adjusting pH and temperature. Thus, the membrane surface can actively degrade a fouling layer on its surface and regain initial permeability. Fouling tests with solutions of protein, oil, and mixtures of both, were performed, and the membrane’s ability to self-clean the fouled surface was characterized. Membrane characterization was conducted by investigation of the immobilized enzyme concentration, enzyme activity, water permeation flux, fouling tests, porosimetry, X-ray photoelectron spectroscopy, and scanning electron microscopy.

## 1. Introduction

**Figure 1:**
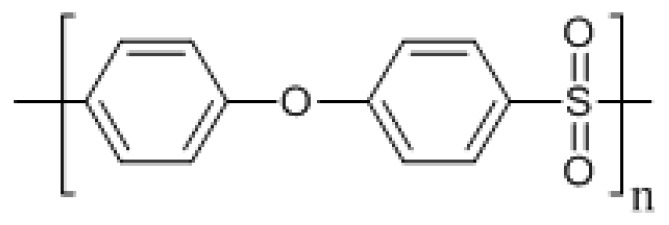
Chemical structure of polyethersulfone (PES).

Diverse modern filtration technologies (e.g., waste water treatment, sterilization filtration, hemodialysis, dairy industry) require the application of tailored polymer membranes [1]. Because of their excellent physical and chemical stability, these polymer membranes are often fabricated from synthetic materials such as polyethersulfone (PES, Figure 1), polysulfone, or polyvinylidene fluoride [2]. However, fouling is a serious problem of membranes made from these hydrophobic polymers. This is often explained by the hydrophobic interactions of the membrane surface with biomolecules or colloids in the filtration mixture resulting in irreversible adsorption, aggregation, and finally, in a reduced filtration performance [3,4,5,6].

To remove the fouling layer, membranes have to be cleaned regularly during the filtration application using strong oxidants such as hypochlorite or citric acid. Commonly, cleaning is performed by treating the membranes with a daily chemically enhanced backwash, a weekly maintenance cleaning with a higher chemical concentration, and an intensive chemical cleaning once or twice a year [7].

To improve the antifouling properties of polymer membranes, many approaches for surface hydrophilization have been reported such as copolymerization or grafting with hydrophilic monomers [8,9,10,11,12,13,14], blending using hydrophilic polymers [15,16,17,18,19,20,21,22,23], and, finally, chemical modification of the membrane polymer [24]. However, most of the presented hydrophilization methods bear serious disadvantages as they are prone to contaminate the eluent by non-covalently bound compounds or by the used initiators/catalysts [25,26].

Previous studies have shown a new electron beam-based immobilization method for directed grafting of hydrophilic small molecules [27,28], polymers [29,30], and even enzymes [31,32,33] to the membrane surface. The irradiation with electrons leads to a simultaneous surface activation of the membrane polymer and covalent immobilization of the desired compounds using an aqueous system. The immobilization of the digestive enzyme trypsin leads to significantly improved antifouling properties. Furthermore, the biocatalytic membrane can be simply “switched on” to actively degrade a fouling layer on the membrane surface and regain the initial permeability [33].

The present study investigates the immobilization of pancreatin on a PES membrane. Pancreatin is a mixture of digestive enzymes including lipase, protease, and amylase which is widely used in food and pharmaceutical industry [34,35,36,37,38,39,40,41,42,43,44]. Immobilized on a membrane surface, this enzyme mixture should provide simultaneous self-cleaning properties towards the three main classes of biomolecules: proteins, lipids, and saccharides. This first study focused on the development of an immobilization method and characterization of the resulting membrane properties regarding enzyme concentration, water permeation flux, scanning electron microscopy, mercury porosimetry, and X-ray photoelectron spectroscopy. The enzymatic activity of lipase and protease was determined and first fouling and self-cleaning studies with solutions of protein, oil, and mixtures of both, were performed. Finally, stability tests of the bioactive membranes in terms of storage time and temperature were performed.

## 2. Experimental Section

### 2.1. Chemicals and Materials

Polyethersulfone membranes (PES, Express Plus, 0.22 µm) were purchased from Millipore (Merck Millipore, Billerica, MA, USA). Pancreatin from porcine pancreas, albumin from bovine serum (BSA, 67 kDa), *N*-α-benzoyl-arginine 4-nitroanilide hydrochloride (BAPNA), dimethyl sulfoxide (DMSO), 1-ethyl-3-(3-dimethylaminopropyl) carbodiimid (EDC), 4-(2-hydroxyethyl)-1-piperazineethanesulfonic acid (HEPES), 4-nitrophenyl palmitate, disodium hydrogen phosphate dihydrat, *N*-hydroxysuccinimid (NHS), *p*-nitroaniline, calcium chloride dihydrate, and phosphate buffered saline (PBS, pH 7.4) were obtained from Sigma Aldrich (St. Louis, MO, USA). 2-Aminoethyl methacrylate hydrochloride (AEMA) was purchased from Acros Organics (part of Thermo Fisher Scientific, Geel, Belgium). Hydrochloric acid and sodium hydroxide were obtained from Merck (Merck Millipore, Billerica, MA, USA). Oil from linseeds (Kunella Feinkost, Cottbus, Germany) used for membrane fouling was purchased from a grocery store. Sodium dodecyl sulfate was obtained from Carl Roth (Karlsruhe, Germany). Bicinchoninic acid (BCA, Pierce, IL, USA) protein assay reagent A + B was provided by Thermo Fisher Scientific (Geel, Belgium). Deionized water was used for preparing all buffer solutions. Unless otherwise stated, water was used in Millipore grade. All chemicals were of analytical grade and used without further purification.

### 2.2. Pancreatin Immobilization

Pancreatin was immobilized after prior electron beam modification of the PES microfiltration membrane. First, the membrane was put into a 0.5 wt % aqueous solution of AEMA followed by irradiation with an irradiation dose of 150 kGy [45]. Irradiation was performed in an N_2_ atmosphere with O_2_ quantities <10 ppm using a home-made electron accelerator. The voltage and the current were set to 160 kV and 10 mA, respectively. The absorbed dose was adjusted by the speed of the sample transporter. The modified membrane was rinsed with water three times per 20 min. In a subsequent reaction, pancreatin was immobilized on the membrane surface. The membrane was immersed into a solution containing 3 wt % pancreatin, 2 mM EDC, and 2 mM NHS in HEPES buffer (100 mM, containing 10 mM CaCl_2_, pH 8) [46]. The membrane was put on a shaker overnight at room temperature and was subsequently rinsed with PBS buffer (pH 7.4) three times per 20 min. Until further usage, the membrane was stored in PBS puffer (pH 7.4).

### 2.3. Membrane Characterization

#### 2.3.1. Scanning Electron Microscopy (SEM)

The surface morphologies of clean and fouled membranes were investigated using an Ultra 55 SEM (Carl Zeiss Ltd., Göttingen, Germany) under magnifications ranging from 1000 to 10,000. The samples were manually cut and subsequently coated with a thin (30 nm) chromium film using the Z400 sputter system from Leybold, Hanau, Germany.

#### 2.3.2. X-ray Photoelectron Spectroscopy (XPS)

The chemical composition was determined using X-ray photoelectron spectroscopy (XPS, Kratos Axis Ultra, Kratos Analytical Ltd., Manchester, UK). Samples of the reference membrane and the modified membrane were investigated.

#### 2.3.3. Mercury Porosimetry

Pore size distribution and porosity of the reference membrane and the modified membrane were determined with a mercury porosimeter (PoreMaster 30, Quantachrome Instruments, Odelzhausen, Germany) to prove that no pore blocking occurred due to the modification. Values of two different samples were averaged.

#### 2.3.4. Contact Angle Measurement

The water contact angle was investigated using a static water contact angle measurements system (DSA 30E, Krüss, Hamburg, Germany) and the sessile drop method. Values of at least three different samples were averaged.

#### 2.3.5. Pancreatin Concentration

The PES membranes modified with pancreatin were analyzed regarding their immobilized enzyme amount using the BCA assay [47]. Therefore, the samples were washed three times with 1 mL of PBS buffer solution (pH 7.4). Then, the BCA reagent was added to the membrane samples and the plate was incubated for 25 min at 37 °C. The plate was then shaken for 5 min at room temperature, the solution was transferred to a new microtiter plate and light adsorption at 562 nm was measured using a microtiter plate reader (Infinite M200, Tecan, Germany). For calibration, seven pancreatin concentrations of 1000, 500, 250, 125, 62.50, 31.25 and 0.00 μg/mL were used.

#### 2.3.6. Protease Activity

The protease activity of the immobilized pancreatin was determined using BAPNA as substrate according to the method of Oliveira et al. [48]. The 0.3 mM substrate solution was prepared by mixing BAPNA, 1 mL DMSO and 9 mL HEPES buffer (100 mM, containing 10 mM CaCl_2_, pH 8). Then, 3.5 mL of the substrate solution was added to the modified and washed membranes in a 12-well microtiter plate. Every well contained four membrane discs (diameter of 1 cm) and a metal grid to fix the membrane discs in their position. Then, the optical absorption of the solution at 405 nm was monitored over a period of 16 h using a microtiter plate reader (Infinite M200, Tecan, Männedorf, Switzerland). The resulting extinctions from the assay were converted into nmol. For calibration, six standards based on a 0.3 mM *p*-nitroaniline stock solution with concentrations of 42.00, 21.00, 10.5, 5.25, 2.62 and 0.00 µg/mL were used.

#### 2.3.7. Lipase Activity

The lipase activity of the immobilized pancreatin was determined as described above, but 4‑nitrophenyl palmitate was used instead of BAPNA. The 0.6 mM substrate solution was prepared by mixing 4-nitrophenyl palmitate, 1 mL DMSO and 9 mL HEPES buffer. Then, 3.5 mL of the substrate solution was added to the modified and washed membranes and the optical absorption of the solution at 405 nm was monitored over a period of 1 h using a microtiter plate reader (Infinite M200, Tecan, Männedorf, Switzerland).

#### 2.3.8. Fouling and Self-Cleaning Experiments

Fouling experiments were performed as described in one of our previous publications [33] and were interpreted according to the water permeation flux. To simulate a long-time application, high starting concentrations of the protein albumin (2 g/L BSA in 50 mM PBS buffer) or linseed oil (0.5 g/L 2 mM SDS solution) were used. In addition, a mixture (1:1) of these solutions was used to show that both foulants can be addressed at the same time. All filtration experiments were performed using a 50 mL stirred cell (Amicon, Merck Millipore, Billerica, USA, active membrane area: 15.9 cm²) in dead end filtration mode. 

**Figure 2:**
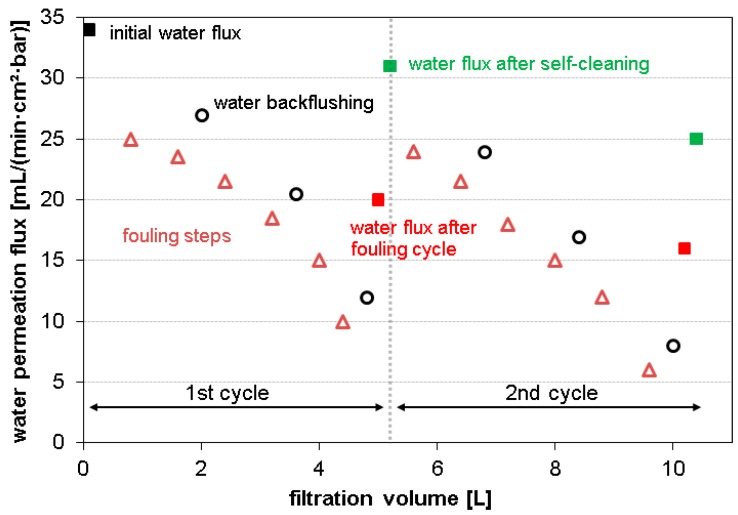
General procedure of fouling experiments using albumin, linseed oil or a mixture of both, and subsequent self-cleaning of the membrane.

For every fouling experiment, the initial pure water permeation flux was measured first by passing 100 mL of water through the membrane and recording the time of flow-through (see Figure 2). Then, six fouling steps (800 mL per step for BSA fouling, 400 mL per step for oil fouling and fouling of the mixture) were performed. The time of flow-through was always recorded for the first 100 mL of every step. After every two steps, a backwash step (100 mL of pure water) was included to remove loosely bound fouling layers. In the end, the pure water permeation flux was determined again. The membrane was immersed into PBS buffer at pH 8 and stored at 37 °C overnight to activate the enzymes. Finally, the membrane was washed three times per 20 min and the pure water permeation flux was measured to evaluate the self-cleaning effect of the membranes.

In addition, fouling cycles with BSA and linseed oil were repeated three times to investigate the possibility of reutilization of the enzymes. Furthermore, membranes modified with pancreatin were stored in buffer solution for ten days at either room temperature or at 4 °C. These membranes were then used to investigate BSA fouling as described above to show that the enzyme modification is stable even after a long period of storage.

## 3. Results and Discussion

### 3.1. Biocatalyst Immobilization on Membrane Surface

To covalently immobilize the enzyme mixture of pancreatin, a standard coupling procedure using NHS/EDC was applied (see Section 2.2) [46]. Since this method requires the presence of free amino groups on the membrane surface, the PES membrane was first modified by electron beam-induced grafting with 2 aminoethyl methacrylate hydrochloride [45]. The functionalized membrane surface then reacts via NHS/EDC with free carboxylic acid groups of the enzyme to form a covalent amid bond. The successful immobilization of pancreatin was confirmed by a XPS and BCA test to determine the enzyme concentration (Table 1). 

XPS results show the appearance of nitrogen on the membrane after immobilization of pancreatin (8.72%). Furthermore, changes in the oxygen and sulfur amount (Ref: O 27.98%, S 3.88%; PES-Pancreatin: O 22.32%, S 1.51%) can be explained with the new formed enzyme layer on top of the membrane surface that covers the polymer structure of the PES membrane. Furthermore, the BCA test resulted in a high concentration of protein (107.4 µg/cm²) after pancreatin immobilization. However, the contact angle of the membrane surface was not significantly changed due to pancreatin immobilization (see Table 1). This can be explained by the fact that the reference PES membrane was already hydrophilized by the manufacturer. Therefore, a contact angle of 58° was determined for the reference membrane, indicating a hydrophilic surface. After pancreatin immobilization, a contact angle of 55° was found. The enzyme mixture on the surface results in a comparable surface hydrophilicity.

**Figure 3:**
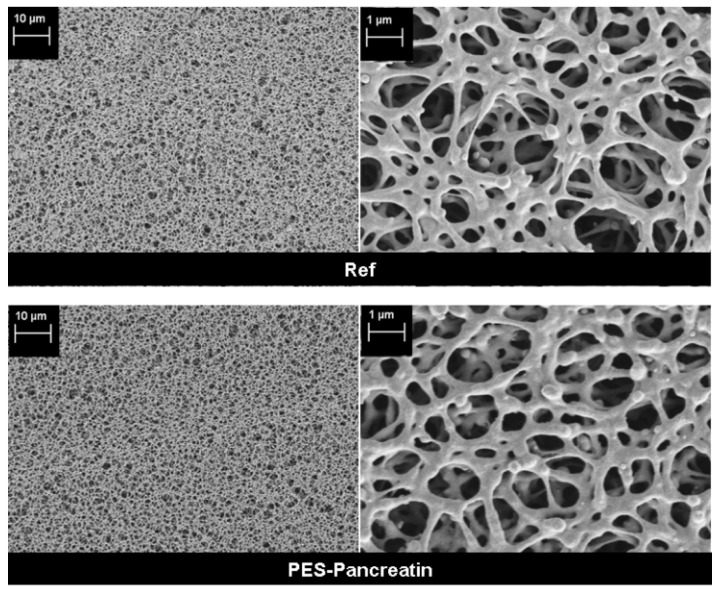
SEM images of membrane surfaces: Reference membrane (**top**) and membrane with immobilized pancreatin (**bottom**).

The physical structure of the membrane was investigated by mercury porosimetry and SEM. No significant change of average pore size and porosity (Table 1) was found after modification. Compared to the pore size of 0.88 µm, the layer of immobilized pancreatin is too thin to affect the pore size or porosity of the membrane. This conclusion is also supported by the SEM images (Figure 3). The membrane structure is not changed. Furthermore, no pore blocking or defects can be detected. Therefore, the modification method seems to be suitable to immobilize the enzyme mixture without changing the physical structure of the supporting membrane.

### 3.2. Fouling and Self-Cleaning

After confirming that the membrane surface exhibits protease and lipase activity in principle as described in Section 2.3.6 and Section 2.3.7, the biocatalytic activity of the modified membrane was evaluated in several fouling experiments. First, the membrane was used for protein/oil filtration (see Section 2.3.8) and the change in pure water flux was monitored. Then, the pancreatin enzymes were activated by immersing the membrane into a buffered solution at 37 °C and pH 8.0 overnight. Under these conditions, the enzymes will catalyze the hydrolysis of the fouling layer and clean the surface. Afterwards, the pure water flux was detected again to evaluate the self-cleaning ability.

**Figure 4:**
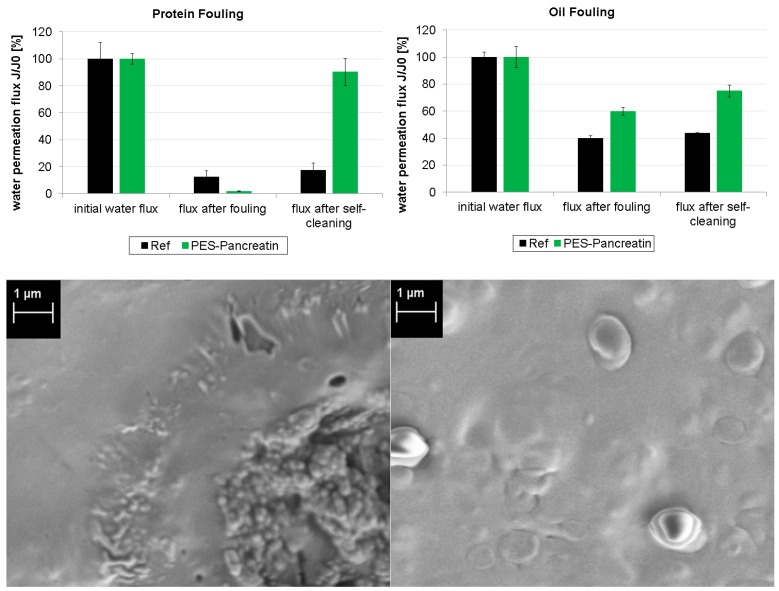
Fouling and self-cleaning properties of the reference membrane and the PES-Pancreatin membrane were characterized by initial water permeation flux, water flux after fouling treatment, and water flux after self-cleaning. Displayed are experiments with protein fouling (**left**, **top**) and oil fouling (**right**, **top**). Furthermore, SEM images of the PES-Pancreatin membrane were recorded after fouling with protein (**left**, **bottom**) and oil (**right**, **bottom**).

Figure 4 displays the results of the first fouling experiments. After the fouling treatment with proteins, the Ref membrane as well as the PES-Pancreatin membrane are significantly blocked since the water flux is decreased to 12% and 2%, respectively. This was confirmed by the respective SEM images that show complete blocking of the membrane pores. After the self-cleaning step, the flux of the Ref membrane does not change (because it does not contain any pancreatin enzymes).

In contrast, the PES-Pancreatin membrane regained 90% of the water permeation flux. This proves that the pancreatic protease was successfully immobilized and actively degrades a protein fouling layer. In case of the oil fouling, the decrease in water permeation due to fouling was less pronounced. The Ref membrane showed a water flux of 40%, and the PES-Pancreatin membrane had a water flux of 60% after oil fouling. However, after the self-cleaning step, the Ref membrane again did not improve the flux performance. In contrast, the PES-Pancreatin membrane regained 75% of its original water flux. This means that the pancreatic lipase enzyme was also successfully immobilized and actively self-cleans the fouled membrane surface.

**Figure 5:**
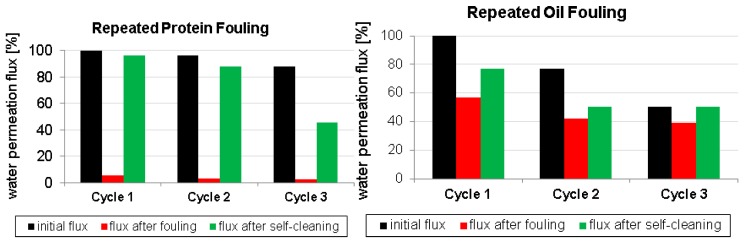
Repeated (three times) fouling and self-cleaning experiments of the PES-Pancreatin membrane.

To evaluate the possibility of repeated self-cleaning, the fouling experiments were then repeated in three cycles (for details see Section 2.3.8 and Figure 2). The results are presented in Figure 5. When the PES-Pancreatin membrane is exposed to protein fouling, the water flux is decreased dramatically after the fouling step. Fortunately, in every self-cleaning step, the fouling layer is significantly cleaned and the water permeation flux increases again. However, after three cycles, the water permeation after self-cleaning is lower compared to the first or second cycle. We used high protein concentrations to simulate heavy fouling. Thus, it is likely that with repeated fouling cycles at one point there will be adsorption of the protein onto the enzymes of the biocatalytic membrane, resulting in decreased water permeation flux, as well as blocking of the active site of the enzymes. A similar trend is found for repeated oil fouling experiments. Although the lipase enzyme is still active after three fouling cycles, the total recovery of water flux by self-cleaning becomes smaller.

**Figure 6:**
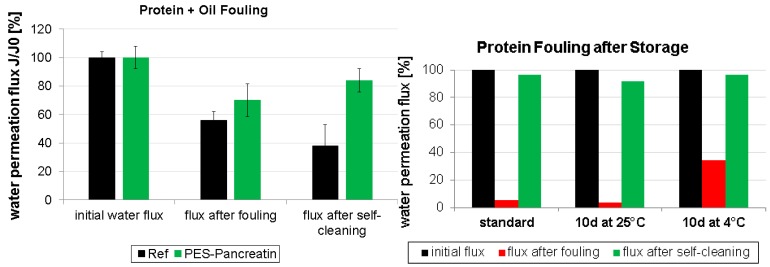
Fouling and self-cleaning experiments using a mixture of protein and oil **(left**) and stability experiments regarding different storage periods and temperatures (**right**).

Finally, fouling was also performed using a combination of protein and oil (Figure 6 left). Comparable to the results after oil fouling, the water flux reduction by fouling was found to be less pronounced. However, self-cleaning of the PES-pancreatin membrane regained 84% of the water flux. It can be assumed that the immobilization of the enzyme mixture is also capable of actively degrading mixtures of fouling compounds such as proteins and oil.

To further investigate the stability of the biocatalytic membrane, different storage conditions were tested (Figure 6 right) before performing protein fouling experiments. After synthesis, the PES-pancreatin membrane was stored for ten days either at 25 or 4 °C and compared to a freshly prepared membrane regarding fouling and self-cleaning properties. The membranes are all highly active after this storage period and show comparable water flux recovery values (higher than 91% in all cases). Interestingly, the effect of fouling was less strong in the case of the membrane that was stored at 4 °C. Here, the water flux was reduced to 34% compared to 5% when stored at 25 °C. This can be explained by an even higher activity of the protease when stored at lower temperatures leading to self-cleaning during the fouling procedure.

Further experiments are necessary to tailor the stability of the used pancreatin enzymes. The activity of the present amylase must be evaluated and more combinations of different fouling reagents should be used to support the hypothesis of a membrane with the ability to generally self-clean its surface after nonspecific fouling.

## 4. Conclusions

The enzyme mixture of pancreatin was successfully immobilized on a PES membrane by a covalent NHS/EDC coupling method. The presence of the enzymes was confirmed by XPS, and BCA tests, respectively. Furthermore, membrane characterization by SEM and porosimetry confirmed that a very thin layer of enzyme is immobilized on the membrane without negatively changing the physical structure of the supporting membrane. 

The activity of protease and lipase present in the pancreatin enzyme mixture was successfully demonstrated by fouling experiments with proteins and oil. After “switching on” the catalytic activity, the membrane surfaces showed impressive self-cleaning and gained a water flux recovery of 90% and 75% after protein and oil fouling, respectively. It was demonstrated that fouling treatments and self-cleaning steps can be repeated several times. Furthermore, a mixture of proteins and oil was also used for fouling of the membrane. A successful self-cleaning was demonstrated in this case, too. Finally, the biocatalytic membranes can also be stored for ten days at 4 or 25 °C without an activity loss. Interestingly, less fouling occurred in the case of the membrane that was stored at 4 °C, indicating a self-cleaning activity during the fouling step. 

This new biocatalytic self-cleaning membrane system will enable efficient and ecological use in filtration applications, avoiding extensive chemical cleaning treatments, and therefore, reduce waste and energy effort. Because the immobilization of pancreatin will equip the membrane with different enzymes, different fouling problems could be addressed simultaneously.

## Figures and Tables

**Table 1:** Results of XPS, BCA, contact angles, and porosimetry investigation of the reference PES membrane (Ref) and after immobilization of pancreatin (PES-Pancreatin).

Sample	Elemental ratio (rel. atom %)				Protein conc.	Contact angle	Average pore size	Porosity
	C 1s	O 1s	N 1s	S 2p	(µg/cm²)	(°)	(µm)	(%)
Ref	68.13	27.98	-	3.88	0.3 ± 0.5	58 ± 2	0.88 ± 0.02	85 ± 2
PES-Pancreatin	67.45	22.32	8.72	1.51	107.4 ± 4.7	55 ± 4	0.89 ± 0.02	86 ± 2
